# Single- and Dual-Task Balance Training Are Equally Effective in Youth

**DOI:** 10.3389/fpsyg.2018.00912

**Published:** 2018-06-06

**Authors:** Benjamin Lüder, Rainer Kiss, Urs Granacher

**Affiliations:** ^1^Division of Training and Movement Sciences, Research Focus Cognition Sciences, University of Potsdam, Potsdam, Germany; ^2^Department of Geriatrics, AGAPLESION Bethanien Heidelberg, Heidelberg University, Heidelberg, Germany

**Keywords:** postural control, cognitive performance, attentional demand, dual-task costs, cognitive interference

## Abstract

Due to maturation of the postural control system and secular declines in motor performance, adolescents experience deficits in postural control during standing and walking while concurrently performing cognitive interference tasks. Thus, adequately designed balance training programs may help to counteract these deficits. While the general effectiveness of youth balance training is well-documented, there is hardly any information available on the specific effects of single-task (ST) versus dual-task (DT) balance training. Therefore, the objectives of this study were (i) to examine static/dynamic balance performance under ST and DT conditions in adolescents and (ii) to study the effects of ST versus DT balance training on static/dynamic balance under ST and DT conditions in adolescents. Twenty-eight healthy girls and boys aged 12–13 years were randomly assigned to either 8 weeks of ST or DT balance training. Before and after training, postural sway and spatio-temporal gait parameters were registered under ST (standing/walking only) and DT conditions (standing/walking while concurrently performing an arithmetic task). At baseline, significantly slower gait speed (*p* < 0.001, *d* = 5.1), shorter stride length (*p* < 0.001, *d* = 4.8), and longer stride time (*p* < 0.001, *d* = 3.8) were found for DT compared to ST walking but not standing. Training resulted in significant pre–post decreases in DT costs for gait velocity (*p* < 0.001, *d* = 3.1), stride length (-45%, *p* < 0.001, *d* = 2.4), and stride time (-44%, *p* < 0.01, *d* = 1.9). Training did not induce any significant changes (*p* > 0.05, *d* = 0–0.1) in DT costs for all parameters of secondary task performance during standing and walking. Training produced significant pre–post increases (*p* = 0.001; *d* = 1.47) in secondary task performance while sitting. The observed increase was significantly greater for the ST training group (*p* = 0.04; *d* = 0.81). For standing, no significant changes were found over time irrespective of the experimental group. We conclude that adolescents showed impaired DT compared to ST walking but not standing. ST and DT balance training resulted in significant and similar changes in DT costs during walking. Thus, there appears to be no preference for either ST or DT balance training in adolescents.

## Introduction

Previously, human postural control has been considered an automatic task that requires minimal cognitive demand. However, research using dual-task (DT) paradigms showed that cognitive resources are needed to control standing (Palluel et al., 2010) and walking (Krampe et al., 2011) in children and adolescents. During everyday activities, adolescents often encounter situations involving the concurrent performance of attention-demanding tasks while standing or walking. For example, they may walk through crowded places or cross busy streets on their way to school while concurrently paying attention to other people, street lights, cars, cell phones. Thus, their attentional capacity has to be adequately divided between the primary (postural) and the secondary (cognitive) task to allow a safe way to school. However, shared capacity may result in performance declines in the primary task, the secondary task, or in both tasks.

In general, numerous original articles (Boonyong et al., 2012; Hung et al., 2013) and review papers (Ruffieux et al., 2015) clearly revealed performance decrements during DT compared to single-task (ST) situations in youth. Based on a systematic analysis of the literature on DT performance during lifespan, Ruffieux et al. (2015) reported slower gait speed, shorter stride length, and larger postural sway in DT compared to ST conditions in youth, young and old adults. More specifically, the authors identified impaired DT balance performance (one-legged stance) particularly in youth (age: <8–13 years) compared to young (age: 19–35 years) and old adults (age: >59 years). It has to be noted though that the authors rated the available evidence in youth as inconclusive (Ruffieux et al., 2015). In children aged 8–9 years, cross-sectional studies (Beurskens et al., 2015, 2016a) also revealed significant decreases in gait velocity, stride length and cadence while walking in DT compared with ST conditions. Within the youth age group, Palluel et al. (2010) compared ST and DT balance performance in 12–15-year-olds versus 17-year-olds. The study revealed larger postural sway and higher sway velocity in the younger age group. Those decrements in walking performance may significantly increase potential risks during ambulation (e.g., when crossing a street and talking to a classmate or looking at a cell phone).

Many studies investigating DT performance calculated dual-task costs (DTC) to describe performance differences between ST and DT conditions in youth (Schaefer et al., 2008; Krampe et al., 2011). DTC yield one single measure rather than utilizing ST and DT performance separately. Positive values indicate deteriorated performance from ST to DT condition that is, declines in the primary postural task and/or the secondary cognitive/motor task during DT compared to ST condition. Negative values on the other hand represent better performances (Somberg and Salthouse, 1982). Previously, the occurrence of DTC have primarily been explained by limited cognitive capacities (Pashler, 1994) or cognitive interference when two tasks share the same processing resources (Wickens, 1984). More recently, concurrent performance models of multitasking have focused on the use of multiple resources (e.g., the “4-D multiple resource model” (Wickens, 2008), “model of *threaded cognition*” (Salvucci and Taatgen, 2011)]. In contrast to single-channel and specifically bottleneck theories, resource models incorporate the idea that the available somewhat limited resources can be scheduled and allocated to specific task processing, i.e., shared between multiple tasks in varying proportions (cf. Fischer and Plessow, 2015).

The model of threaded cognition (Salvucci and Taatgen, 2011), for example, accounts for dual-task interference patterns by adducing multiple resource constructs within perceptual modalities (cf. Wickens, 2008). The main premise of the model is that multiple threads of cognitive processing can be active at the same time. However, multitask interferences occur when (multiple) threads or goals are simultaneously active and require the same cognitive resource at the same time. Consequently, one thread must wait and its performance will be adversely affected (cf. Salvucci and Taatgen, 2011).

Difficulties in allocating attentional resources to two tasks or the inability to manage additional cognitive demands caused by limited information processing capacity may provoke DTC in postural control. In adolescents, deficits in postural control have primarily been attributed to immaturity of the visual and vestibular systems which represent two major afferent systems that contribute to postural control (Hirabayashi and Iwasaki, 1995; Steindl et al., 2006). Thus, there is a need to elucidate whether DT balance performance can be improved through adequate training regimes in youth.

There is ample evidence on the general effectiveness of balance training on balance performance in youth as indicated in randomized controlled trials (Granacher et al., 2010a; Pau et al., 2012; Donath et al., 2013) and recent systematic reviews (Gebel et al., 2018). In an attempt to extend the findings of Granacher et al. (2010a) who demonstrated that balance training is suitable to enhance ST postural control, specifically designed intervention programs during PE may have the potential to improve postural control not only in ST but also in DT situations. However, to the authors’ knowledge, there are currently no studies available that examined the specific effects of ST versus DT balance training in youth, especially concerning dual-task performance. Hence, our rationale is largely grounded on studies using other cohorts (i.e., seniors). Of note, the general effects of balance training on balance performance are well documented in seniors (Lesinski et al., 2015). In terms of the specific effects of ST versus DT balance training in old adults, Silsupadol et al. (2009a) reported that DT but not ST balance training resulted in improved DT balance performance in the form of faster habitual gait speed while performing an arithmetic interference task. However, the specific effects of ST versus DT balance training on DT performance have not yet been examined in youth. Hence, this study design follows the previously introduced approach from Silsupadol et al. (2009a) in geriatric populations and translates it from seniors to youth. In addition, there is evidence from adult studies that DT balance training may be superior to ST balance training in improving DT performance (Wollesen and Voelcker-Rehage, 2014). Thus, in order to decrease adolescents’ DTC in balance performance, DT balance training might be an effective tool to improve the capacity to perform a motor and cognitive task concurrently by minimizing the cognitive overload.

Therefore, the main objectives of this study were (i) to examine static and dynamic balance performance under ST and DT conditions in healthy adolescents, (ii) to study the effects of traditional ST versus DT balance training in adolescents on static and dynamic balance under ST and DT conditions (i.e., standing/walking while concurrently performing an arithmetic subtraction task). With reference to the relevant literature (Boonyong et al., 2012; Hung et al., 2013; Wollesen and Voelcker-Rehage, 2014; Ruffieux et al., 2015), we expected impaired standing/walking performance during DT compared to ST balance performance in adolescents. We further hypothesized that DTC in static and dynamic balance is particularly reduced following DT balance training. In accordance with the principle of training specificity (Behm, 1995), we expected larger effects for static (i.e., standing) compared with dynamic (i.e., walking) balance because both balance training protocols primarily consisted of static balance exercises during standing on stable (i.e., gym floor) and unstable surfaces (i.e., balance pads) while balancing only (ST group) or while performing secondary tasks during the performance of balance exercises (i.e., DT group).

## Materials and Methods

### Participants

Twenty-eight healthy adolescents were recruited from a primary school located in the state of Brandenburg (City of Potsdam), Germany. Their characteristics are displayed in **Table 1**. Participants had no known neuromuscular or orthopedic disorders that might have affected their ability to perform the experiment. None had participated in research on posture or cognition within the preceding 6 months. Fourteen out of 28 enrolled participants were active members in sports clubs and 19 participants reported to be regularly engaged in self-organized physical activities (cycling, home workouts, or running). An a priori power analysis using two groups and a repeated measure ANOVA design yielded a total sample size of *N* = 28 (effect size = 0.25, α = 0.05), with an actual power of 0.8 (critical *F*-value = 4.23). Effect size was based on a study that examined the effects of balance training on postural control in adolescents (Granacher et al., 2010a). The Human Ethics Committee at the University of Potsdam approved the study protocol (reference number: 04/2014). Before the start of the study, each participant and their parents/legal guardians read, concurred, and signed written informed consent. All procedures were conducted according to the latest version of the Declaration of Helsinki.

**Table 1:** Participants’ characteristics (mean ± standard deviation).

	Total (*N* = 28)	ST-BAL (*n* = 13)	DT-BAL (*n* = 15)
Sex (m/f)	13/15	6/7	7/8
Age (years)	13.3 ± 0.5	13.0 ± 0.3	13.4 ± 0.6
Body height (cm)	156.0 ± 7.1	155.9 ± 5.4	155.0 ± 9.0
Body mass (kg)	43.8 ± 8.1	41.5 ± 6.3	45.9 ± 9.9
BMI (kg/m^2^)	18.0 ± 3.1	17.0 ± 2.1	19.1 ± 3.9
Physically active (%)	67.9	61.5	73.3
Membership in sport clubs (%)	50	46.2	53.3
School grades (range)			
German	1 (1–3)	1 (1–2)	1 (1–3)
Math	2 (1–4)	2 (1–3)	2 (1–4)
English	1 (1–4)	1 (1–3)	1 (1–4)


**BMI, body-mass-index; ST-BAL, single-task balance training group; DT-BAL, dual-task balance training group; f, female; m, male; school grades are displayed as median (range).**

### Data Registration

Testing procedure included the assessment of static and dynamic postural control in ST and DT situations. During ST conditions, only the respective motor or cognitive task had to be performed, whereas during DT conditions, participants were asked to concurrently perform an attention-demanding interference task (i.e., to recite out loud serial subtractions by 3, starting from a random number between 300 and 900). When DT methodology was used, participants were instructed to do both tasks as best as they can and thus give equal priority to both tasks in order to create real-life conditions. A similar procedure has been applied previously (Granacher et al., 2010b). The order of all experimental conditions was counterbalanced across participants and the assessors were blinded regarding group allocation.

### Assessment of Static Postural Control

Static postural control was assessed using a three-dimensional force plate (Leonardo Mechanograph GRFP, Novotec Medical, Germany). The force plate consisted of eight sensors with a sampling rate of 800 Hz per sensor and registered center of pressure (CoP) displacements in medio-lateral (ML) and anterior-posterior (AP) direction. Participants were instructed to stand on their dominant leg (as assessed by the lateral preference inventory) (Coren, 1993). The non-supporting limb was flexed 45° at the knee, hands were placed akimbo and gaze fixated at a cross on a nearby wall. The length of standing trials was standardized to 30 s each. Excellent intra- (ICC = 0.97; 95% CI: 0.91–0.99) and intersession (ICC = 0.94; 95% CI: 0.84–0.98) reliability were reported for the one-legged stance (Muehlbauer et al., 2011). High interrater (ICC = 0.87–0.99) and test–retest (ICC = 0.59–0.99) reliability for the one-legged stance was reported in children (Atwater et al., 1990). Total CoP displacements were computed according to the following formula: $CoP[mm]=\sqrt{{CoP}_{{AP}^{2}}+{CoP}_{{ML}^{2}}}$. CoP_AP_ represents CoP displacements in anterior–posterior and CoP_ML_ represents CoP displacements in medio-lateral direction. In addition, *CoP velocity*, indicating the total distances covered by the CoP divided by the duration of the sampled period and *sway area*, representing the ellipse area covered by the trajectory of the CoP were calculated (Schubert and Kirchner, 2014). Participants performed one trial in ST and one trial in DT condition.

### Assessment of Dynamic Postural Control

Gait performance was registered using a 10-m instrumented walkway (OptoGait, Microgate, Bolzano, Italy). The OptoGait-System is an opto-electrical measurement device consisting of light-transmitting and -receiving bars. Each bar is 1 m in length and composed of 96 light emitting diodes transmitting to an oppositely positioned bar. With a continuous connection between two bars, any break in connection can be measured and timed. Participants’ spatial and temporal gait characteristics were registered at 1,000 Hz. The OptoGait-System demonstrated high discriminant (error: <2%) and concurrent validity (ICC: 0.93–0.99) with a validated electronic walkway (GAITRite^®^-System) for the assessment of spatio-temporal gait parameters in healthy participants (Lienhard et al., 2013). Excellent intra-class correlation coefficients [ICC (2, 1) = 0.929–0.998] and coefficients of variation (CV_ME_ = 0.32–11.30%) were previously reported (Lee et al., 2014). Excellent test-retest reliability [ICC (3, 1) = 0.785–0.952] of the gait parameters measured by the OptoGait-System was demonstrated as well (Lee et al., 2014). *Gait velocity* was defined as distance covered per second during one stride. *Stride length* was defined as linear distance between successive heel contacts of the same foot, and *stride time* as time between the first contacts of two consecutive footfalls of the same foot. Participants performed one walking trial in ST and one trial in DT condition.

### Assessment of Secondary Task Performance

For the assessment of secondary task performance, we registered the number of accurate calculations during DT conditions. If a participant miscalculated, the false calculation was noted. When correctly continuing the serial 3 subtraction, only one error was noted (no consequential errors were registered). Additionally, participants were asked to perform as many calculations as possible in 30 s while seated (i.e., ST condition). To compare secondary task performance across conditions (i.e., seated, standing, walking), calculations per second were used for our statistical analyses.

### Balance Training Programs

In a quasi-experimental approach, two school classes were randomly assigned to either perform ST balance training (ST-BAL) or DT balance training (DT-BAL). Thus, the class and not the single participant was our unit of analysis in order to minimize transfer effects through the exchange of training experiences between intervention and control participants within one class. Both groups performed a progressive balance training program for 8 weeks. The training session consisted of a ∼5 min child-oriented warm-up consisting of small games and a 15 min balance training program. Participants were supervised by an expert on balance training together with the PE teacher of the two classes so that the participant to supervisor ratio amounted to 1 (supervisor): 7 (participants). Both supervisors provided feedback on exercise technique and task execution. Training sessions were integrated into the regular PE lessons (total duration: 135 min/week) and conducted during the warm-up period. Each warm-up session lasted 20–30 min. Following balance training, both groups conducted the same curriculum during PE classes. Both balance training protocols primarily consisted of static balance exercises during standing on stable (i.e., gym floor) and unstable surfaces (i.e., balance pad). Training progression was realized by periodically increasing the demand of the balance exercises. Training progressed from static bipedal (e.g., leaning forward/backward/side-ways) to static unipedal exercises (e.g., one-legged stance). The difficulty level was gradually increased by instructing the participants to perform the exercises with or without the help of their arms, their eyes opened or closed and/or on unstable training devices (i.e., soft mats, ankle disks, balance boards, air cushions). Occasionally, a few dynamic balance exercises (e.g., twisting jumps, stabilizing balance in one-legged stance after high knee running) were implemented. The ST-BAL group performed balance exercises only, whereas the DT-BAL group additionally integrated primarily attention-demanding cognitive (e.g., counting backward, naming objects, spelling, etc.) and/or occasionally motor interference tasks (e.g., juggle, roll a ball backward/forward with the free leg, etc.). Different secondary tasks were included in the training protocol that were not part of testing. The rationale behind this approach was to conduct a child-oriented and enjoyable training program for youth (cf. Wälchli et al., 2017) and to examine whether potential transfer effects occur. According to established dose-response relations in balance training, participants conducted four sets of 20 s for each exercise with 1 min rest between sets (Lesinski et al., 2015). Both legs were alternately exercised during one-legged stance.

### Statistical Analyses

Data are presented as mean values and standard errors. One way analyses of variances (ANOVA) with repeated measure on Condition (ST vs. DT) were used to identify baseline differences in static and dynamic postural control between conditions. Participants’ performances in ST compared to DT condition were analyzed separately for each measure using baseline values of the total group (*N* = 28). For further group analyses and to quantify participants’ ability for executing two tasks concurrently, we calculated DTC for each participant and each outcome measure, according to the established formula (Somberg and Salthouse, 1982): $DTC\left[\%\right]=\left(\frac{ST-DT}{ST}\right)\times 100$, where “ST” represents participant’s performance in single-task condition and “DT” represents participant’s performance in dual-task condition. Positive DTC values indicate DT-related performance impairments and negative DTC values indicate improved performance during DT as compared to ST conditions. Separate 2 (Time: pre, post) × 2 (Group: ST-BAL, DT-BAL) ANOVAs with repeated measure on Time were computed to examine performance changes following training and univariate ANOVAs with repeated measure on Time (pre, post) were used to examine potential learning effects in the cognitive interference task. Effect sizes were determined by calculating Cohen’s *d*-values (Cohen, 2013). All analyses were conducted using Statistical Package for Social Sciences (SPSS) version 23.0 (IBM Corp., New York, United States) and significance levels were set at α = 5%.

## Results

**FIGURE 1:**
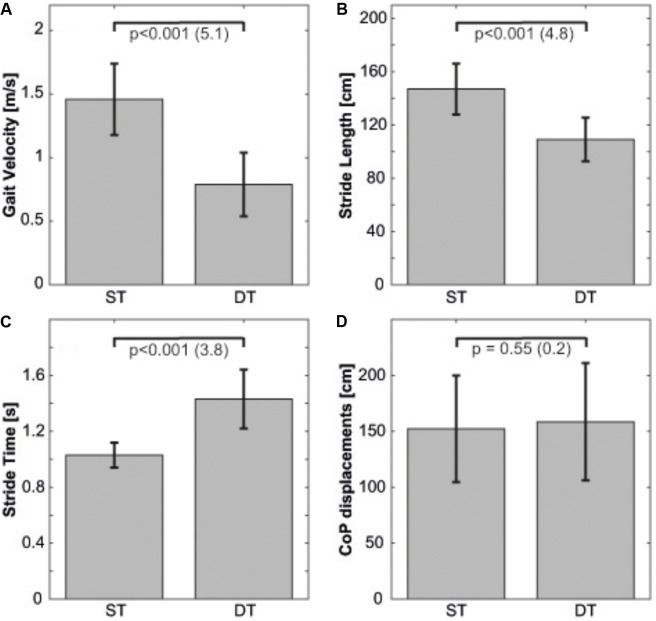
Dynamic and static postural control during single-task and dual-task conditions at baseline, displayed separately for **(A)** gait velocity, **(B)** stride length, **(C)** stride time, and **(D)** total CoP displacements. Error bars represent the respective standard errors. Values in brackets represent Cohen’s *d*. CoP, center of pressure; DT, dual-task; ST, single-task.

All participants received treatment as allocated. Overall, there were no statistically significant between group baseline differences in measures of age, anthropometrics (i.e., height, mass, BMI), and performance (e.g., static/dynamic balance performance, arithmetic secondary task) (all *p* > 0.05). Twenty-eight participants completed the balance training programs and none reported any training- or test-related injury. **Figures 1A–D** illustrate participants’ baseline ST and DT performances. All analyzed walking parameters significantly deteriorated during DT compared to ST condition. That is, gait velocity (*p* < 0.001, *d* = 5.1) and stride length (*p* < 0.001, *d* = 4.8) decreased while stride time increased (*p* < 0.001, *d* = 3.8). **Table 2** describes results of measured ST and DT walking parameters at baseline. For standing (one-legged stance) and secondary task performance, none of the examined parameters was affected during DT condition (all *p* > 0.05, *d* = 0.2–0.5).

**Table 2:** Outcome measures [ANOVA with within-factor Condition (ST vs. DT)].

	Means ± *SD*		*p*-value (*d*)
		
	ST	DT	
Gait velocity (m/s)	1.45 ± 0.3	0.77 ± 0.3	*p* < 0.001 (5.1)
Stride length (cm)	146.1 ± 18.6	108.3 ± 15.9	*p* < 0.001 (4.8)
Stride time (s)	1.03 ± 0.1	1.54 ± 0.5	*p* < 0.001 (3.8)


**ST, single-task condition; DT, dual-task condition; Figures in square brackets represent Cohen’s d.**

### DTC in Dynamic and Static Postural Control Pre and Post Balance Training

**FIGURE 2:**
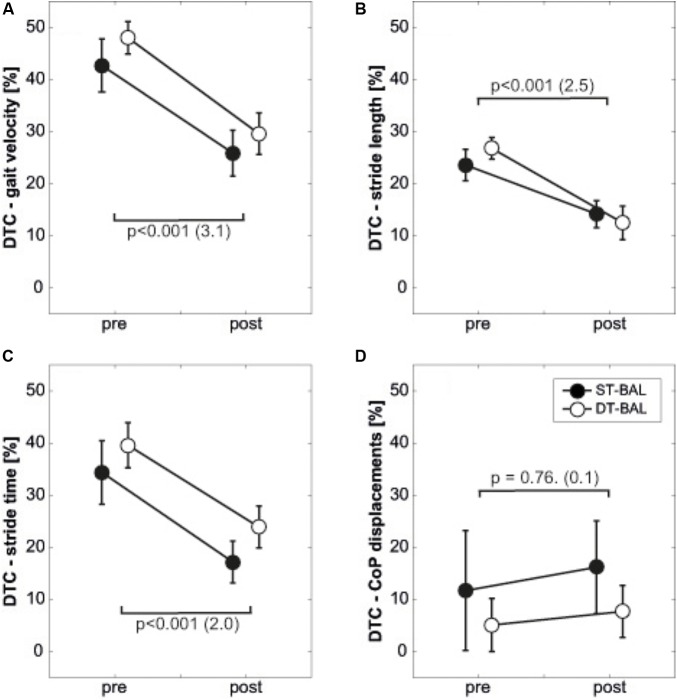
Dual-task costs pre and post intervention, displayed separately for **(A)** gait velocity, **(B)** stride length, **(C)** stride time, and **(D)** total CoP displacements. Error bars represent the respective standard errors; CoP, center of pressure; DTC, dual-task costs; DT-BAL, dual-task balance training group; ST-BAL, single-task balance training group; *p*-values indicate the main effect of Time. Values in brackets represent Cohen’s *d*.

**Tables 3A,B** describe pre- and post-intervention results and the corresponding ANOVA outcomes for parameters of postural control. **Figures 2A–D** display participants’ DTC (note: for static postural control only DTC of total CoP displacements are shown). ANOVA yielded a significant main effect of Time for each gait parameter. That is, DTC decreased by 38–39% (*p* < 0.001, *d* = 3.1) for gait velocity, by 40–53% (*p* < 0.001, *d* = 2.5) for stride length, and by 40–50% (*p* < 0.001, *d* = 2.0) for stride time. No significant effects of Group and Time × Group interactions were observed for any of the examined gait parameters (all *p* > 0.05, all *d* < 0.5).

**Table 3:** Outcome measures (ANOVA with repeated measures on Time).

	ST-BAL (*n* = 13)			DT-BAL (*n* = 15)			*p*-value (*d*)		
			
	Pre	Post	Δ	Pre	Post	Δ	Time	Group	Group × Time
(A) Dynamic balance performance									
DTC – gait velocity (%)	42.7 (5.1)	25.9 (4.4)	-39	48.0 (3.1)	29.6 (3.9)	-38	< 0.001 (3.1)	0.39 (0.3)	0.69 (0.2)
DTC – stride length (%)	23.6 (3.0)	14.2 (2.6)	-40	26.8 (2.1)	12.5 (3.2)	-53	< 0.001 (2.5)	0.81 (0.1)	0.19 (0.5)
DTC – stride time (%)	34.4 (6.1)	17.2 (4.0)	-50	39.6 (4.3)	23.9 (3.9)	-40	< 0.001 (2.0)	0.22 (0.5)	0.92 (0)
(B) Static balance performance									
DTC – CoP displacement (%)	11.7 (11.5)	16.2 (8.9)	+38	5.1 (5.1)	7.7 (4.9)	+51	0.76 (0.1)	0.31 (0.4)	0.67 (0.2)
DTC – CoP velocity (%)	12.6 (11.7)	16.2 (8.9)	+28	6.2 (4.2)	5.8 (5.2)	-6	0.96 (0)	0.28 (0.4)	0.57 (0.2)
DTC – sway area (%)	26.6 (13.1)	10.7 (16.3)	-60	13.1 (14.6)	14.9 (15.2)	+14	0.63 (0.2)	0.59 (0.2)	0.40 (0.3)
(C) Secondary task performance									
DTC – calculations (stand) [%]	-10.1 (12.7)	2.6 (6.5)	+74	-16.6 (8.6)	-27.8 (10.9)	-68	0.85 (0.1)	0.14 (0.6)	0.33 (0.4)
DTC – calculations (walk) [%]	5.5 (16.3)	4.8 (8.0)	-12	8.3 (6.5)	-1.0 (9.2)	-112	0.98 (0)	0.78 (0.1)	0.28 (0.4)


**Values represent means (standard error). Figures in square brackets represent Cohen’s d. No group baseline differences were detected (all p > 0.05); CoP, center of pressure; DTC, dual-task costs; DT-BAL, dual-task balance training group; ST-BAL, single-task balance training group.**

Regarding static postural control, our statistical analyses did not detect significant main effects of Time (all *p* > 0.05, *d* = 0–0.2) or Group (all *p* > 0.05, *d* = 0.2–0.4), nor significant Time x Group interactions (all *p* > 0.05, *d* = 0.2–0.3) for any of the examined parameters.

### Secondary Task Performance Pre and Post Balance Training

**FIGURE 3:**
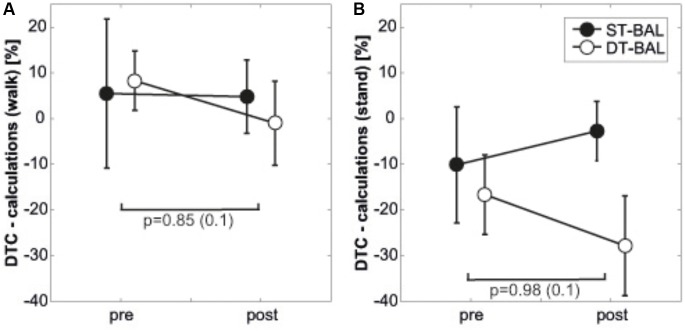
Dual-task costs for secondary task pre and post intervention, displayed separately for **(A)** walking, **(B)** standing. Values in brackets represent Cohen’s *d*; DTC, dual-task costs; DT-BAL, dual-task balance training group; ST-BAL, single-task balance training group.

Analysis of performance in the secondary task while sitting showed a significant and large main effect of Time (*p* = 0.001; *d* = 1.47) and a significant large sized Time × Group interaction (*p* = 0.04; *d* = 0.81). No significant main effects of Group were detected for all examined variables (*p* = 0.30; *d* = 0.41). Whereas the dual-task training group achieved 0.28 correct calculations/s during baseline testing and 0.30 correct calculations/s during post-testing (+7.1%), the single-task training group improved significantly from 0.36 correct calculations/s to 0.40 calculations/s (+11.1%). Neither significant main effects of Time (*p* = 0.478; *d* = 0.27) or Group (*p* = 0.149; *d* = 0.56) nor Time × Group (*p* = 0.446; *d* = 0.29), Group × Condition (*p* = 0.582; *d* = 0.21) or Time × Condition (*p* = 0.305; *d* = 0.40) interactions during standing were found. Participants’ DTC in secondary task performance during standing and walking are illustrated in **Figures 3A,B** and the respective ANOVA outcomes are displayed in **Table 3C**. The analysis yielded no significant main effects of Time (both *p* > 0.05, *d* = 0–0.1), Group (both *p* > 0.05, *d* = 0.1–0.6) or Time × Group interactions (both *p* > 0.05, both *d* = 0.4) for any of the examined parameters.

## Discussion

The present study examined differences in static and dynamic postural control during ST and DT conditions in adolescents aged 12–13 years. We additionally compared the effects of traditionally applied ST as compared to DT balance training on DTC in measures of dynamic (i.e., gait velocity, stride length, stride time) and static (i.e., total CoP displacement, CoP velocity, sway area) postural control and on secondary task performance (i.e., number of accurate calculations). The main findings of this study can be summarized as follows: (i) performances in walking but not standing deteriorated in DT compared to ST condition, (ii) both training regimes resulted in significant changes in measures of DTC during walking but not standing, and (iii) irrespective of the training regime, neither significant main effects of Time and Group nor significant Time × Group interactions were detected for (DTC in) secondary task performance.

### ST vs. DT Performance at Baseline

Results showed that walking was affected when adolescents performed a concurrent arithmetic task. Gait velocity (ST: 1.45 ± 0.3 m/s; DT: 0.77 ± 0.3 m/s) and stride length (ST: 146.1 ± 18.6 cm; DT: 108.3 ± 15.9 cm) decreased and stride time (ST: 1.03 ± 0.1 s; DT: 1.54 ± 0.5 s) increased during DT compared to ST walking. These findings are consistent with previous studies investigating DT vs. ST performance in adolescents (Hung et al., 2013; Ruffieux et al., 2015). In general, the magnitude of the observed decrease in gait velocity in our study is higher than the changes found in a previous study (Boonyong et al., 2012), where adolescents aged <16 years decreased their gait velocity by 4.5% when walking while concurrently conducting an auditory Stroop task. In the present study, adolescents reduced their gait velocity by 0.6 m/s (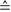
29%), indicating that the cognitive interference effects were substantial. Deficits in DT performance of adolescents might be explained by not fully developed structures (i.e., visual and vestibular systems) within the central nervous system (Riach and Hayes, 1987). More specifically, Hirabayashi and Iwasaki (1995) postulated that the proprioceptive system already matures between ages 3 and 4, while the visual system still develops until age 15. These findings were confirmed by Steindl et al. (2006).

With regard to DT balance performance, Palluel et al. (2010) argued that adolescents reach adult-like performance at the age of 14–15 years. Of note, our participants’ mean age was 13.3 years. At this age, the postural control system is not yet fully matured (Woollacott and Shumway-Cook, 1990).

Findings from imaging studies provided evidence that the prefrontal cortex which is associated with executive functions and DT performance (Szameitat et al., 2002) is not fully developed at age of 14–16 years (Arain et al., 2013) as there is a developmental mismatch in brain maturation, with subcortical regions maturing during adolescence, whereas the prefrontal cortex does not reach a similar level of maturity until adulthood (Somerville et al., 2010; Mills et al., 2014). The (dorsolateral) prefrontal cortex plays a critical role for the regulation and processing of complex cognitive (mostly in executive functions) and motor tasks (Diamond, 2000; Liang et al., 2016). In a recent study, Beurskens et al. (2016b) examined the underlying neural correlates of single- and dual-task walking. Beurskens et al. (2016b) registered neural activation in frontal, central, and parietal brain areas using a mobile 64 channel EEG system. They found that average activity in alpha and beta frequencies was significantly modulated during both cognitive (i.e., participants were asked to respond to a low-pitched tone by pressing a button and inhibit their response to a high-pitched tone) and motor interference (i.e., participants held two interlocked sticks in front of their body which were not supposed to touch each other) walking conditions in frontal and central brain regions. More specifically, lower alpha activity in frontal brain areas was found when walking while concurrently performing the cognitive interference and the motor interference task. This is indicative of an increased cognitive load in the prefrontal cortex during dual-task walking (Beurskens et al., 2016b). The authors concluded that impaired motor performance during dual-task walking is mirrored in neural activation patterns of the brain, which complies with established cognitive theories arguing that DT situations overstrain cognitive capabilities, resulting in motor performance decrements (Beurskens et al., 2016b).

Thus, a decrement in performance during DT situations can most likely be observed due to limited cognitive capacity (i.e., “central overload”) (Pashler, 1994). According to the single-bottleneck theory, the cognitive processes involved in maintaining balance and calculating could only proceed sequentially due to structural capacity limitations. This ultimately resulted in performance decrements (i.e., DTCs) especially in task two in the sequence. However, the notion of structural capacity limitations for central processing stages (bottleneck) in multi-task situations has been debated intensively in recent years (e.g., Logan and Gordon, 2001; Navon and Miller, 2002; Fischer and Plessow, 2015). The central question revolves around whether cognitive processes related to different tasks proceed only sequentially (one at a time), or can operate in parallel (simultaneously). In summary, it has been argued that parallel and serial processing of multiple tasks are not mutually exclusive and that shifting between more parallel and more serial task processing critically depends on the conditions under which multiple tasks are performed (cf. Fischer and Plessow, 2015). Hence, another theory should be taken into consideration.

Under the assumption of the multiple resource theory, division of (cognitive) capacity resources is possible and parallel processing can occur. According to this theory, DTCs arise when the processing of different task components require the same limited resources. The multiple-resource model of attention proposed by Wickens (1984) and the 4-D multiple resource model (Wickens, 2008), respectively, appear to be well-suited to provide detailed information regarding the occurrence of DT deficits in adolescents. The models essentially state that two tasks are most likely to interfere when they share the same pool of cognitive resources. Walking requires central and visual processing; subtracting numbers requires central as well as verbal processing. In other words, if two tasks are concurrently conducted with the primary task demanding postural control and the secondary task requiring cognitive processing, decrements in performance are likely to occur when task demands exceed cognitive capacities (Krampe et al., 2011; Beurskens et al., 2016a). Alternatively, applying the model of threaded cognition (Salvucci and Taatgen, 2011), the DTCs we observed could be explained similarly. In our case, the explanation would comprise that interferences occurred since the two threads or goals “maintaining balance or walking speed” and “correctly solving as many of the arithmetic tasks as possible” were active simultaneously. Moreover, both threads required (at least partly) the same resource at the same time, namely central processing. This adversely affected the performance of the thread that had to wait. For standing performance, no DT-related decrements were found in our study, indicating that the balance task might not have been sufficiently demanding to cause DT-related deficits. This assumption is supported by the fact that secondary task performance (i.e., number of correct calculations) during the standing task remained similar during DT as compared to ST situations. Similarly, for secondary task performance during walking, no DT-related losses in performance were observed.

### Performance Changes Following ST and DT Balance Training

Previous studies showed that ST balance training is suitable to improve balance performance in adolescents (Granacher et al., 2010a; Pau et al., 2012). That is, 4 weeks of ST balance training (three sessions per week on unstable training devices) integrated into PE lessons significantly reduced postural sway. This reduction was not evident in an active control group (i.e., performing generic exercises during warm-up) (Granacher et al., 2010a). Similarly, 6 weeks of ST balance training (18 sessions for 20–30 min each) integrated in regular volleyball training significantly decreased total CoP displacements during bipedal and unipedal stance in 12-year-old adolescents compared to an active control group (i.e., attending regular volleyball training) (Pau et al., 2012). However, none of the studies examined ST balance training effects on DT performance in adolescents.

Following ST balance training, DTC in measures of walking improved in our study. That is, DTC decreased for gait velocity (-47%), stride length (-43%), and stride time (-50%). Similarly, DTC of gait velocity (-41%), stride length (-55%), and stride time (-38%) decreased following DT balance training. There is no study available that scrutinized the effects of ST and DT balance training on DT balance performance in adolescents. Thus, our findings have to be compared with results originating from studies with older cohorts. DT balance training has been shown to improve performance during DT situations in older adults (cf. Wollesen and Voelcker-Rehage, 2014 for a review). Adolescents still show maturational deficits in their motor-cognitive performance (Ruffieux et al., 2015) while older adults are in a state of age-related functional decline (Oberg et al., 1993). Thus, it is plausible to argue that similar adaptations following DT balance training can be expected in adolescents and seniors.

Silsupadol et al. (2009a) examined the effects of an individualized 4-week (three times per week for 45 min. each) DT compared to ST balance training on walking in older adults aged >65 years. DT balance training included walking while counting backward, naming objects, or spelling words backward while ST balance training included walking exercises only. Results showed that ST and DT balance training were suitable to significantly improve dynamic balance performance (i.e., walking speed) (Silsupadol et al., 2009b) and center of mass position (Silsupadol et al., 2009a) during ST conditions. Yet, only DT balance training improved performance in DT walking conditions. These findings are in line with a recent review by Plummer et al. (2015) examining, among others, the effects of DT motor training on DTC in older adults. The authors found that DT training regimes resulted in significant improvements in DT gait speed, thus decreasing DTC. They concluded that DT motor training improved DT walking performance by increasing the speed at which individuals walk in DT conditions. This finding is in accordance with the principle of training specificity which denotes that the applied exercises during training should closely mimic the demands of sport-specific or everyday tasks (Behm, 1995). Concurring with this observation, Wollesen and Voelcker-Rehage (2014) showed evidence that improvements in DT performance are more pronounced following DT compared to ST balance training. Of note here, Wollesen and Voelcker-Rehage (2014) examined healthy old adults in their systematic review.

In our study, walking performance was significantly better following both, ST and DT balance training. A number of methodological reasons may account for the observed differences in study findings between Wollesen and Voelcker-Rehage (2014) and our study. While Wollesen and Voelcker-Rehage (2014) conducted their study in older adults, we examined adolescents aged 12–13 years. Given the different age group and differences in (included) experimental/study design between our study (adolescents; quasi-experimental design) and the studies used in Wollesen and Voelcker-Rehage (2014) systematic review (older adults; RCTs) the differences in the described findings seem to be explainable. In this regard, it appears to be plausible to argue that similar adaptations following DT balance training can be expected in adolescents and seniors. However, this needs to be verified in future studies. Thus, the differences in findings between our study and the mentioned systematic review may be explained by the fact that there are indeed great differences between the respective age groups after all and/or by the limitations of our (single) study (see below). For many years, the control of walking has primarily been seen as an automatic process. Today, it is well-known that attentional resources are necessary to effectively stabilize the body during standing and walking (Woollacott and Shumway-Cook, 2002). It has been shown that ST balance training modifies cortical plasticity (Taubert et al., 2010, 2011) and excitability (Taube et al., 2007; Taube, 2012). In fact, following 2 weeks of balance training [i.e., standing on a moveable platform (stabilometer)] increased gray matter volume in young adults in frontal and parietal regions of the brain (Taubert et al., 2010). Moreover, these authors found that white matter volume increased in the same spatial and temporal pattern. Over the 6 weeks balance training period, Taubert et al. (2010) further demonstrated that initial gray matter changes in sensorimotor-related regions decreased in the later learning phase, while gray matter in the prefrontal cortex continuously increased. These authors interpret their findings as the initial challenge of learning a complex motor skill and an important characteristic for entering later learning stages (Taubert et al., 2010). These results are indicative of training-induced modifications in central processing mechanisms following ST balance training. This assumption is supported by Taube et al. (2007), who found reduced cortical excitability and spinal reflex activity (Taube et al., 2008) following ST balance training. These authors hypothesized that ST balance training and the accompanied improvements in motor performance result in adaptations on the subcortical level of the brain (i.e., in basal ganglia and cerebellum). This hypothesis was supported by findings from Taubert et al. (2011) who reported increased gray matter volume in prefrontal and supplementary-motor areas and additionally, increased activity in subcortical brain regions following ST balance training. While structural changes in gray matter and functional connectivity alterations were most prominent during the first 3 weeks of training, changes in fronto-parietal functional connectivity and the underlying white matter structure developed gradually over the course of the 6 weeks of training (cf. Taubert et al., 2011). According to these authors, it appears that ST balance training induces a shift in activation from cortical to subcortical areas (cf. (Taube, 2012) for a review).

Similarly, DT balance training might induce improved task coordination skills when two tasks have to be performed simultaneously. In general, deficits in DT performance arise if an overload in cognitive capacities occurs (Pashler, 1991) or when two tasks share cognitive/sensory modalities and processing resources (Wickens, 1984). The efficacy of DT balance training might be based on an efficient integration and coordination of two concurrent tasks. During DT balance training, specific DT situations play a crucial role in the training process, which results in an improved performance in these particular tasks. Thus, ST and DT balance training in adolescents might free up central processing capacities and cognitive resources that can then be used to adequately adapt to DT situations while walking.

On the other hand, static postural control (i.e., one-legged stance) and secondary task performance in our study were not affected following ST and DT balance training. The former finding is in contrast to previously published studies in children (Donath et al., 2013) and adolescents (Granacher et al., 2010a; Pau et al., 2012). Granacher et al. (2010a) were able to show that 4 weeks of ST balance training during PE lessons resulted in significantly reduced postural sway in adolescents. However, the absence of improvements in static postural control in our study can primarily be seen as a result of the non-existent DT-related impairments at baseline (cf. **Figure 1D**). However, it was still surprising to see neither significant main effects of Time or Group nor Time × Group, Group × Condition or Time × Condition interactions during standing. We hypothesize that the secondary task might have been too easy for our participants which is why we could not detect interference during standing. This finding is supported by the fact that performance in the secondary task while standing improved following training (pre: 0.32 ± 0.16 correct calculations/s; post: 0.38 ± 0.18 calculations/s) while balance performance declined. The absence of improvements on secondary task performance following training in our study resembles previous findings. In a recent systematic review, Plummer et al. (2015) found that six out of nine studies on the effects of physical exercise interventions (ST/DT balance training, cardiovascular and strength training, multicomponent exercise program, DT treadmill walking) on DT walking performance in older adults reported no significant change in DT performance for the cognitive tasks. To explain the absent effects on cognitive task performance during DT, aspects of task similarity and training specificity have to be taken into consideration. Participants in the DT training group explicitly (but not exclusively) trained cognitive tasks (e.g., calculations or spelling) during DT balance training which is why they were used to these kind of tasks (task similarity). This in turn led to a higher level of automaticity in task execution. Thus, more resources were available for the other, non-cognitive tasks (i.e., maintaining postural control) (Agmon et al., 2015). Consequently, participants of the DT training group improved in postural control rather than cognitive performance following training. Similarly, the ST balance training in the ST training group led to a higher level of automaticity in task execution of the postural control task which in turn resulted in the improvements in DT performance (training specificity). Training-induced improvements in postural control in the form of increased task automatization may have allowed participants to better perform the cognitive tasks by using the gained resources and capacities that were previously needed to adequately control posture (Kramer et al., 1995).

### Limitations

Four potential limitations of this study warrant discussion. First, no passive control group was included in this study. The inclusion of a passive control group is impossible in a school setting, as we cannot expect students and physical education teachers to stop conducting a warm-up program at the beginning of PE lessons. Also, preventing one class from conducting a warm-up program without performing balance exercises was not suitable since the development of postural control is a major part of the syllabus at this stage of PE. However, our aim was not to evaluate general effects of balance training in adolescents. It has previously been shown that balance training is effective and feasible in a school setting (Granacher et al., 2010a) and suitable to improve ST balance performance in adolescents (Granacher et al., 2010a; Pau et al., 2012). Thus, a passive control group was not needed in our study design. We wanted to specifically elucidate the effects of ST versus DT balance training in youth. A second limitation of our study is the implementation of only one trial during ST and DT condition to register standing/walking performance. This limitation was based on our experimental setting and its limitations. We performed all experiments during regular PE classes and thus only a limited period of time was available to conduct all needed measurements. However, if a trial failed, the measurement was repeated to ensure one valid trial per condition for each participant. However, the use of one trial in the one-legged stance and OptoGait 10 m walkway test setting appears to be justified given the excellent reliability values that were reported previously (Atwater et al., 1990; Muehlbauer et al., 2011; Lee et al., 2014). Another limitation of this study may be possible effects of task prioritization on the balance or cognitive performance under DT condition (e.g., see Broeker et al., 2018). However, previous studies (e.g., Wehrle et al., 2010) found that there are no statistically significant differences, neither in balance performance nor in cognitive performance, when instructed to prioritize task one, two or both equally. As this phenomenon seems to be relatively robust (cf. Siu and Woollacott, 2007; Schaefer et al., 2008; Wehrle et al., 2010), we assume that prioritization did not play a significant role in our study. Lastly, we used a group-based training approach limiting the adaptation of training contents to the individual needs of the participants. However, we chose to use the group-based setting since it resembled commonly established training or exercise protocols during PE classes. Typically, physical education is group-based in school settings and we did not want to alter those established structures.

## Conclusion

Adolescents suffer from impaired balance performance while walking during DT compared to ST conditions. This supports the theory that maintaining postural control and solving cognitive tasks (e.g., subtracting numbers) require similar cognitive processing (cf. (Wickens, 1984) and that DT-related performance decrements occurred when task demands exceed cognitive capacities (cf. Pashler, 1991). Further, we conclude that both, ST and DT balance training resulted in significant changes in youth DT walking performance. Lastly, we could not detect any significant effects on DTC in secondary task performance and that is irrespective of the applied training regime.

In conclusion and with regard to the results of our study, there appears to be no preference for either ST or DT balance training in healthy adolescents.

## Author Contributions

All authors listed have made substantial, direct and intellectual contributions to the work, and approved it for publication.

## Conflict of Interest Statement

The authors declare that the research was conducted in the absence of any commercial or financial relationships that could be construed as a potential conflict of interest.
